# Spatiotemporal distribution and meteorological factors of hemorrhagic fever with renal syndrome in Hubei province

**DOI:** 10.1371/journal.pntd.0012498

**Published:** 2024-11-04

**Authors:** Hang Li, Rui Yang, Xuhua Guan, Xiaobo Huang, Honglin Jiang, Liangfei Tan, Jinfeng Xiong, Mingjun Peng, Tianbao Zhang, Xuan Yao

**Affiliations:** Hubei Provincial Center for Disease Control and Prevention, Wuhan, China; University of Queensland & CSIRO Biosecurity Flagship, AUSTRALIA

## Abstract

**Background:**

Hemorrhagic fever with renal syndrome (HFRS) is a vital rodent-borne disease, and poses a serious public health threat in Hubei province. We aimed to explore the spatiotemporal distribution of HFRS in Hubei province during 2005–2022, and the effects of meteorological factors.

**Methods:**

Data on HFRS cases at the county level in Hubei province during 2005–2022 were obtained from the Chinese Center for Disease Prevention and Control Information System. The monthly meteorological data at the city level was extracted from the China Meteorological Data Sharing Service System from 2016 to 2020. Descriptive analyses, joinpoint regression model, spatial correlation analyses, Geodetector model and autoregressive integrated moving average (ARIMA) model were conducted to investigate the epidemic characteristics, temporal trend, spatial distribution, influencing factors of HFRS and predict its trend.

**Results:**

A total of 6,295 cases were reported in Hubei province during 2005–2022, with an average incidence of 6/1,000,000. Most cases were males (74.52%) and aged 40–69 years (71.87%). The monthly HFRS cases showed two seasonal peaks, which were summer (May to June) and winter (November to December). The HFRS incidence remained fluctuating at a low level during 2005–2015, followed an increasing trend during 2015–2018, and then decreased during 2018–2022. Hotspots were concentrated in the center of Hubei province in all 3 periods, including Qianjiang, Tianmen and some counties from Xiangyang, Jingmen and Jingzhou cities. The distribution of HFRS had a positive association with wind speed, while a “V”-shaped correlation with mean temperature, with an explanatory power of 3.21% and 1.03% respectively (both *P* <0.05). The ARIMA model predicted about 1,223 cases occurred in the next 3 years.

**Conclusions:**

HFRS cases showed seasonal fluctuation and spatial clustering in Hubei province. Central plain areas showed high risk of HFRS. Wind speed and mean temperature had significant effects on the transmission of HFRS in Hubei province. The results alert health authorities to conduct disease-climate surveillance and comprehensive prevention strategies, especially in high-risk counties.

## Author summary

A retrospective analysis of surveillance data was conducted to explore the epidemic characteristics and spatiotemporal dynamic distribution of HFRS in Hubei province from 2005 to 2022. The effects of meteorological factors on HFRS incidence were then explored by the Geodetector model. The HFRS incidence remained fluctuating at a low level during 2005–2015, followed an increasing trend during 2015–2018, and then decreased during 2018–2022. HFRS cases were concentrated in the center of Hubei province, with a transmission to southern areas. It is necessary to strengthen surveillance, rodent control and vaccination in these high-risk areas, to prevent the occurrence of outbreaks. Wind speed and mean temperature were significantly associated with HFRS distribution in Hubei province. The information is helpful for the exploration of early warning systems.

## 1. Introduction

Hemorrhagic fever with renal syndrome (HFRS) is a vital vector-borne disease, caused by *Hantavirus*. The major reservoir and infectious source are rats, such as *Rattus norvegicus* and *Apodemus agrarius* in China [1]. Transmission of the virus from rats to human is mainly through close contact with the secretions of infected rodents, or contaminated materials and inhalation of aerosolized urine or faeces [2]. Most patients show clinical symptoms typically including fever, headache, abdominal pain, hemorrhagic tendency, acute renal syndrome, and so forth [3].

HFRS has been reported worldwide, and China is the most affected country, with about 30,000 reported cases every year, accounting for almost 90% of global cases [4]. In Hubei province, the first HFRS case was identified in Wuhan city in 1957 [5]. After that, the disease gradually spread to most counties. The most serious outbreak occurred in 1983, with 23,943 cases reported. A series of preventive strategies have been taken, including vaccine injection and rodent control, leading to a significant decline in HFRS incidence [6]. However, studies reported that there was a slight increase in HFRS incidence during 2015–2017 [7]. Besides, some areas are still affected by high risk of HFRS infection, for example Yicheng, Zhongxiang, Qianjiang, Tianmen and so on [7,8]. It has remained a public health threat in Hubei province.

Previous studies have investigated the temporal and spatial distributions of HFRS. For example, there were 3 studies that explored the spatiotemporal pattern of HFRS in Hubei province during 1980–2009 [5], 2005–2014 [7] and 2010–2017 [9] respectively. However, recent HFRS cases were not taken into consideration. Epidemiological characteristics of HFRS present obviously temporal variations. Study on the long-term epidemiology and dynamics of the HFRS, taking into account recent HFRS cases, will help to understand the disease comprehensively, which is still lacking in Hubei province.

Climate factors are reported to influence the epidemiology and transmission of HFRS, by affecting rodent populations and opportunities for human-rodent interaction [10]. Precipitation, temperature, humidity and wind speed, which could effectively represent the climate characteristics [11], were indicated as the most important elements influencing the endemic intensity of HFRS [10]. It’s widely accepted that these factors may differentially affect the risk of HFRS in different areas [12,13]. A few studies have been conducted in Hubei province, and the results were not completely consistent. Ge L et al. found that the risk of HFRS was positively associated with average humidity, while not significantly related to average temperature or total rainfall by conducting Spearman’s rank correlation analysis in Hubei province [7]. He then observed that HFRS incidence was negatively associated with average temperature in the seasonal difference-geographically and temporally weighted regression model [14].

In this study, we aimed to delineate the epidemic characteristics and spatiotemporal dynamic distribution of HFRS in Hubei province by a retrospective analysis of surveillance data, and quantify the effects of meteorological factors on HFRS by an ecological study, including mean temperature, precipitation, wind speed and relative humidity. A better understanding of epidemiology and risk factors of HFRS is required to contain the possibility of future epidemics.

## 2. Materials and methods

### 2.1. Data sources

Data on HFRS cases in Hubei Province from January 1, 2005 to December 31, 2022 were derived from the Chinese Center for Disease Prevention and Control Information System. All HFRS cases were diagnosed by exposure history, clinical symptoms and laboratory inspection, based on Diagnostic Criteria for HFRS classification. Corresponding resident population data for each county was obtained from the Hubei Statistical Yearbooks during 2005–2022. The city-scale’s meteorological factors, namely monthly mean temperature, precipitation, wind speed and relative humidity, were extracted from the China Meteorological Data Sharing Service System from January 1, 2016 to December 31, 2020.

### 2.2. Statistical analysis

#### Epidemic characteristic analysis

The epidemic characteristics of reported HFRS cases including distributions in terms of year, month, sex, age groups and occupation were described by descriptive statistics. All analyses were performed by IBM SPSS Statistics 27.0.1.

#### Joinpoint regression model

Temporal trend of HFRS incidence in Hubei Province was estimated by joinpoint regression model (JPR) [15]. The default maximum number of joinpoints is associated with the number of data points. As suggested in the Joinpoint help system (https://surveillance.cancer.gov/help/joinpoint/setting-parameters/method-and-parameters-tab/number-of-joinpoints), a maximum number of three points was set. The annual percentage of change (APC) was estimated for each segment. A positive value for APC informs an increased trend of HFRS incidence; otherwise, it shows a decreased trend. The JPR model was performed by Joinpoint Regression Program 4.7.0.0 from the US National Cancer Institute (https://surveillance.cancer.gov/joinpoint/).

#### Spatial correlation analysis

Spatial auto-correlation analysis has been widely employed to measure the dependence considering the location and distance in geographic data [16]. The global spatial autocorrelation analysis describes the overall degree of spatial auto-correlation in the specified region. The global Moran’s *I* index is one of the principal indicators, ranging from -1 to 1. A zero value shows a random spatial distribution. A positive index value indicates a clustered pattern; otherwise, it informs a dispersed spatial pattern. Then local indicators of spatial association (LISA) was conducted to examine the spatial correlation with neighborhood accurately by calculating local Maran’s *I* index. A positive value of local Maran’s *I* index indicates similar values in adjacent areas, including high-high and low-low clusters. The negative values are considered as spatial outliers, including high-low (a high value surrounded by low values) outliers and low-high (a low value neighboring to a high value) outliers. The spatial correlation analysis was performed using ArcGIS 10.8.2.

#### Geodetector model

Geodetector model was then conducted to measure the spatial stratified heterogeneity (SSH) of HFRS during 2016–2020, and identify the driving meteorological factors [17]. Geodetector model is emerging in recent years. The model assumes that the spatial patterns will be consistent if X is associated with Y. The factor detector was conducted in the current study. The tool could calculate *q* value to quantity the extent to which a factor X explains the SSH of Y as follows:

$$q=1-\frac{1}{N\sigma^{2}}\sum_{h=1}^{L}N_{h}\sigma_{h}^{2}$$

Where *h (h* = 1, 2, …*L*) is the stratification of independent variable X. The continuous variables must be discretized according to experience or statistical characteristics of the data. In the *GD* package in R, the continuous variables could be categorized based on natural breaks, sample quantiles, equal interval, geometrical interval or standard deviation. The number of layers was set between 3 and 4 in our study. The model with the maximum *q* value, which was the optimal discretization method, was selected for subsequent analysis. The *N* and *N*_*h*_ are the number of samples of the study area and the stratum *h*, respectively. σ^2^ and σ_h_^2^ represent the variances of HFRS risk in the study area and the stratum *h*, respectively. The *q* represents that the factor X explains 100%*q of the HFRS incidence, ranging from 0 to 1. The Geodetector model was performed by the *GD* package in R (version 4.2.3).

#### Time trend forecasting

Finally, we forecasted the incident HFRS cases in the next 3 years by autoregressive integrated moving average model (ARIMA). The ARIMA model is expressed as ARIMA (*p*, *d*, *q*) (*P*, *D*, *Q*), where *p* represents the autoregressive order; *d* represents the difference order; *q* represents the moving average order; and the *P*, *D*, *Q* represent corresponding seasonal orders. The parameters are determined by autocorrelation function (ACF) plot, stationarity test, and partial autocorrelation function (PACF) plot, respectively [18]. In the current study, we used the “expert modeler” function in SPSS for automatic selection. Mean absolute error (MAE), mean absolute percentage error (MAPE) and root mean squared error (RMSE) were used to measure the goodness-of-fit. The model was performed by IBM SPSS Statistics 27.0.1.

## 3. Results

### 3.1. General characteristics of HFRS epidemic in Hubei province

**Fig 1:**
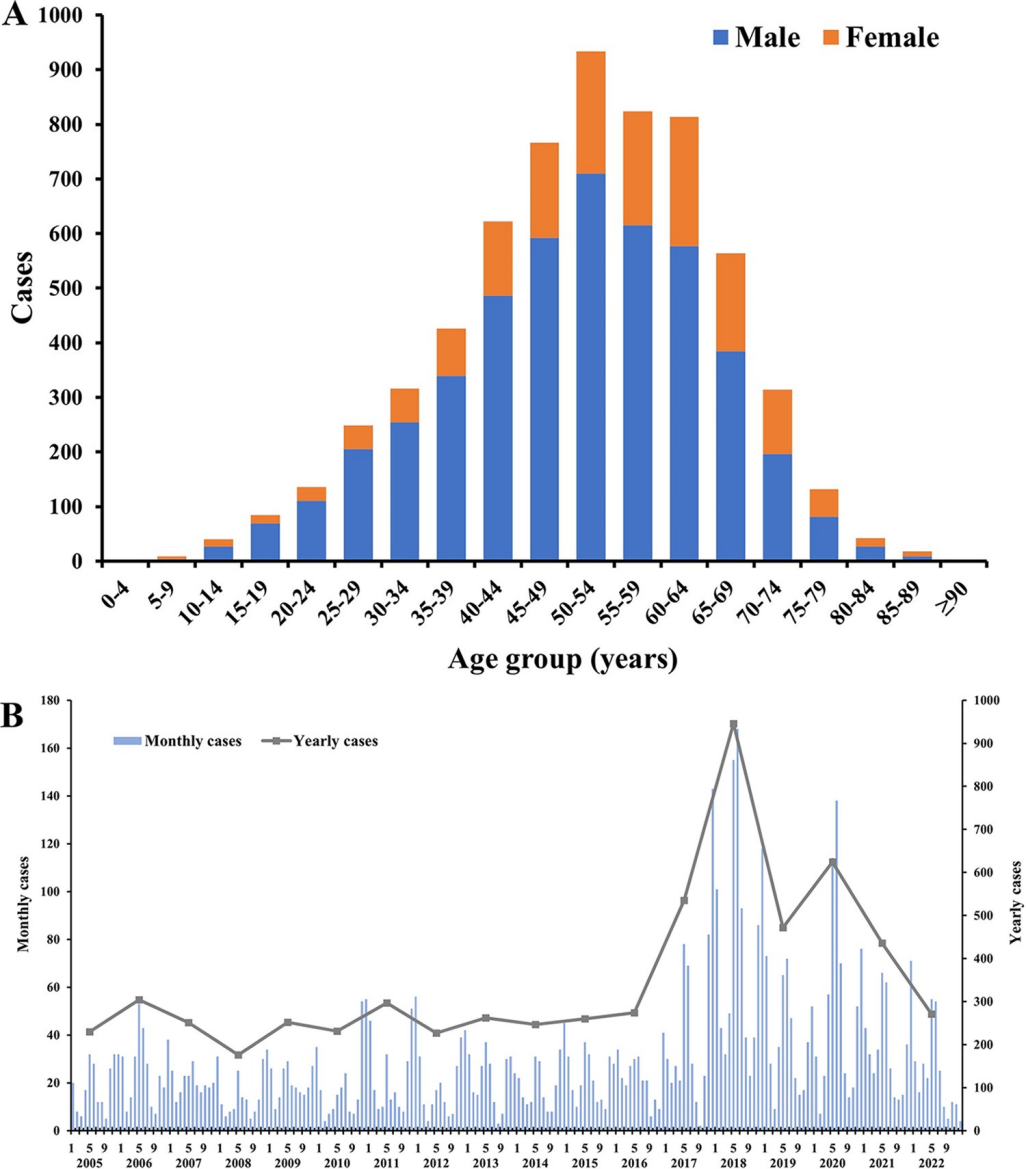
General characteristics of HFRS in Hubei province (2005–2022). (A) Sex and age-group distributions of HFRS; (B) Temporal distribution of HFRS.

A total of 6,295 HFRS cases were reported in Hubei province during 2005–2022, with an average annual incidence of 6/1,000,000 persons, ranging from 3 to 15.8 per 1,000,000 persons. **Fig 1A** shows the distributions of HFRS cases in terms of sex and age groups. Most of the cases (74.52%) were males, with a male-to-female ratio of 3:1. And the difference was observed across different age groups. The age of HFRS cases ranged from 2 to 97 years old, and the median (interquartile range) age was 52 (42–61) years old. 71.87% of cases were notified in 40–69 years. In terms of the vocation distribution, 74.38% of HFRS cases were farmers.

**Fig 2:**
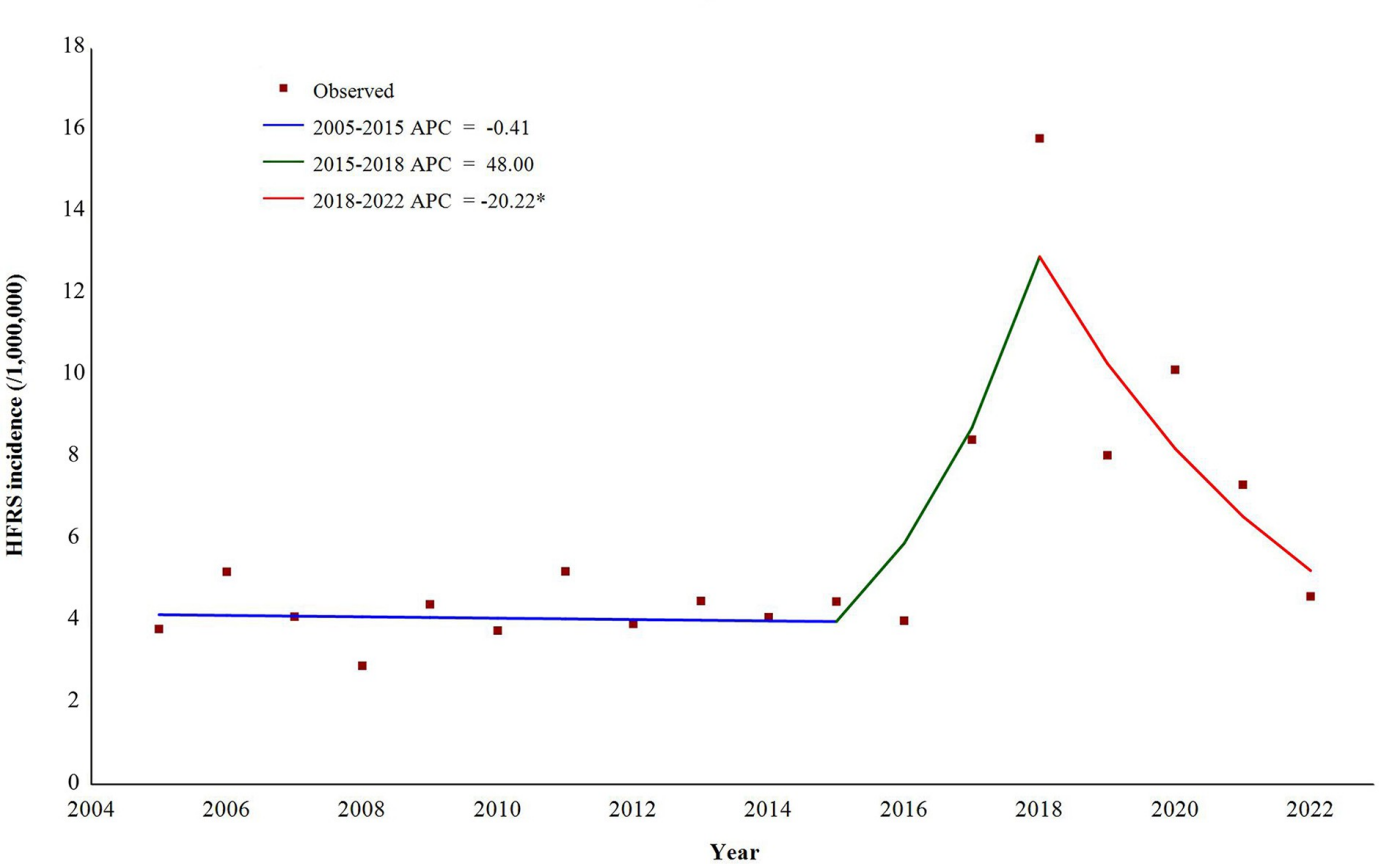
Trend variation on the HFRS incidence at the provincial level, 2005–2022. Note: * indicates that the annual percent change is significant (*P* < 0.05).

**Fig 1B** displays the monthly and yearly HFRS cases during 2005–2022 in Hubei province. The monthly distribution of HFRS cases displayed seasonal fluctuations, with two peaks in summer (May to June) and winter (November to December) separately. As for the long-term temporal trend, the JPR model informed that there were 2 segmental points, placing 3 phases for the trend of HFRS incidence during 2005–2022 (**Fig 2**). The incidence remained fluctuating at a low level, with a slight decreasing trend during 2005–2015 (APC = -0.41%, *P* = 0.85), and followed an increasing trend during 2015–2018 (APC = 48.00%, *P* = 0.19). And then there was a significant decreasing trend during 2018–2022 (APC = -20.22%, *P* = 0.03).

**Fig 3:**
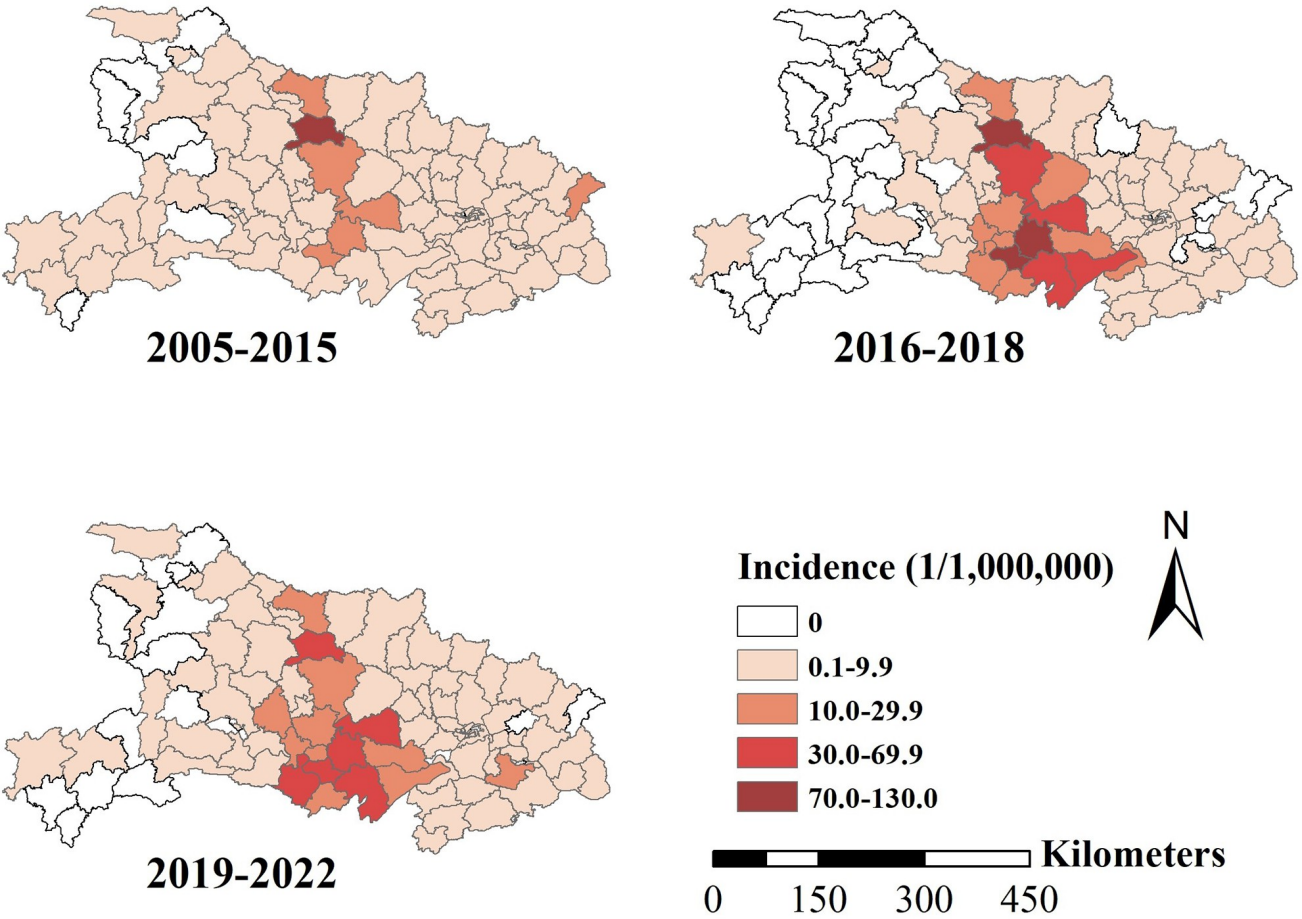
The average incidence of HFRS at the county level in Hubei province (2005–2022). The base layer of the map of Hubei province was acquired from the Hubei Provincial Platform for Common Geospatial Information Services (https://hubei.tianditu.gov.cn/standardMap).

The annual incidence of HFRS ranged from 0 to 227.9 per 1,000,000 persons at the county level for the 18-year period. Based on the 3 phases divided by JPR model, we explored the spatial distribution of incidence of HFRS at the county level separately. The HFRS cases were concentrated in the central areas of Hubei province, with an increasing trend. Yicheng exhibited the highest average HFRS incidence during 2005–2015. During 2016–2018, Qianjiang and Jiangling also exhibited the highest HFRS incidence, and Zhongxiang, Tianmen, Jianli and Honghu had the second highest incidence. The incidences of these counties, except Gong’an, declined during 2019–2022 (**Fig 3**).

### 3.2. Spatial auto-correlation

**Table 1** displays the results of global spatial auto-correlation analysis on HFRS incidence in the 3 phases. All Moran’s *I* indices were positive, ranging from 0.15 to 0.40. The results indicated that the HFRS incidence of all counties in the Hubei province showed significant cluster spatial patterns. Besides, the trend for spatial aggregation was strengthening.

**Table 1:** Spatial auto-correlation analysis for incidence of HFRS in Hubei province (2005–2022).

Study period	Moran’s *I*	Z-score	*P* value	Distribution pattern
2005–2015	0.1479	3.7357	<0.001	Cluster
2016–2018	0.2704	5.2864	<0.001	Cluster
2019–2022	0.4032	7.3956	<0.001	Cluster

**Fig 4:**
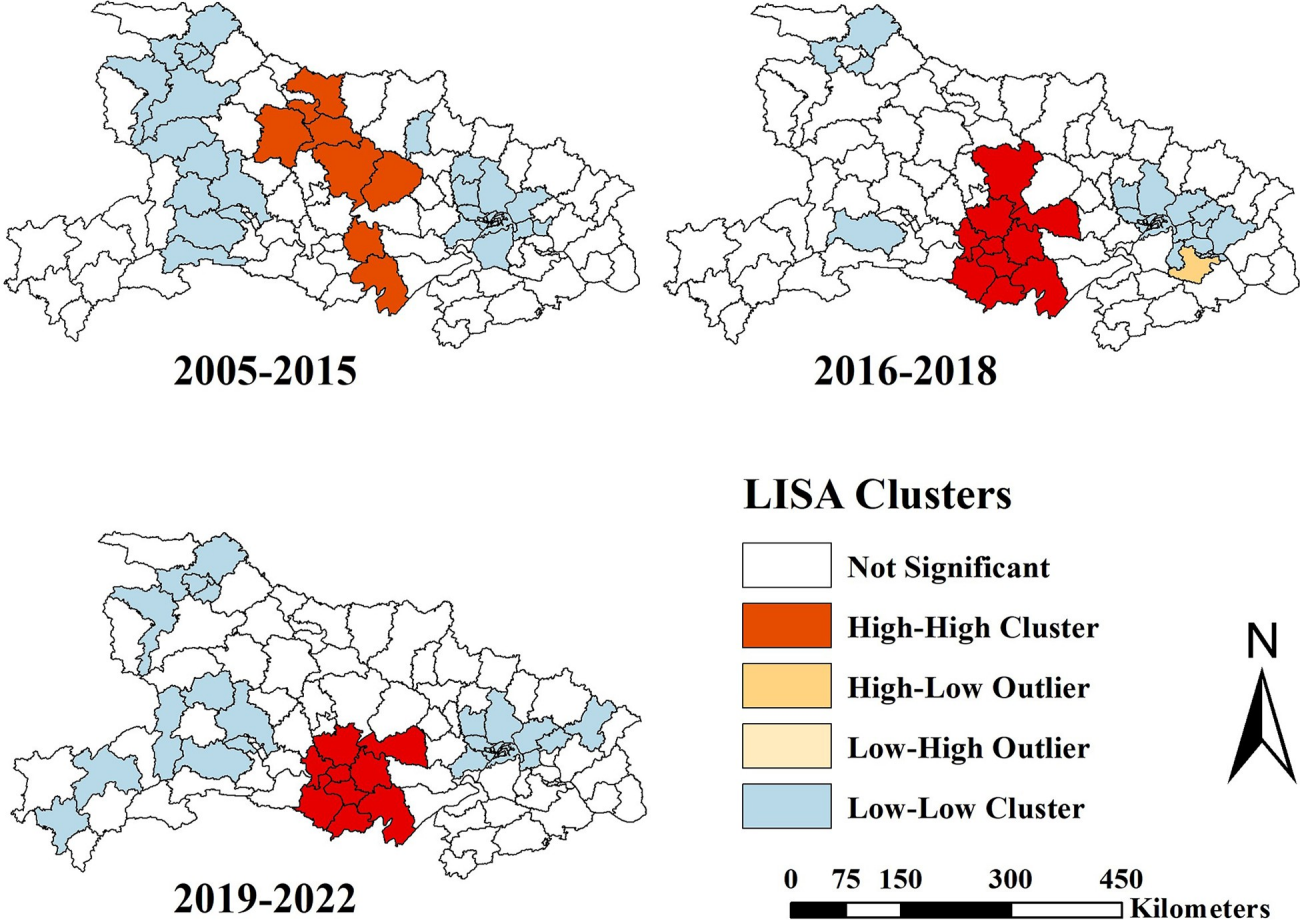
LISA cluster map of HFRS in Hubei province, 2005–2022. The base layer of the map of Hubei province was acquired from the Hubei Provincial Platform for Common Geospatial Information Services (https://hubei.tianditu.gov.cn/standardMap).

**Fig 4** illustrates the dynamic distributions of LISA cluster map during the 3 phases. On the whole, the hotspots were distributed in the center of the Hubei province, with a transmission to southern areas. During 2005–2015, some districts from Xiangyang city constituted primary HFRS endemic areas, including Xiangzhou, Xiangcheng, Nanzhang, Yicheng counties. Besides, Zhongxiang, Jingshan counties from Jingmen city, Jianli county from Jingzhou city and Qianjiang city were also identified as hotspots. The hotspots transmitted to Qianjiang, Tianmen, Jingmen and Jingzhou cities in 2016–2018. Moreover, the hotspots spread to more counties in Jingzhou city. In the subsequent period, the hotspots were similar, apart from Zhongxiang.

### 3.3. Geodetector model

**Fig 5:**
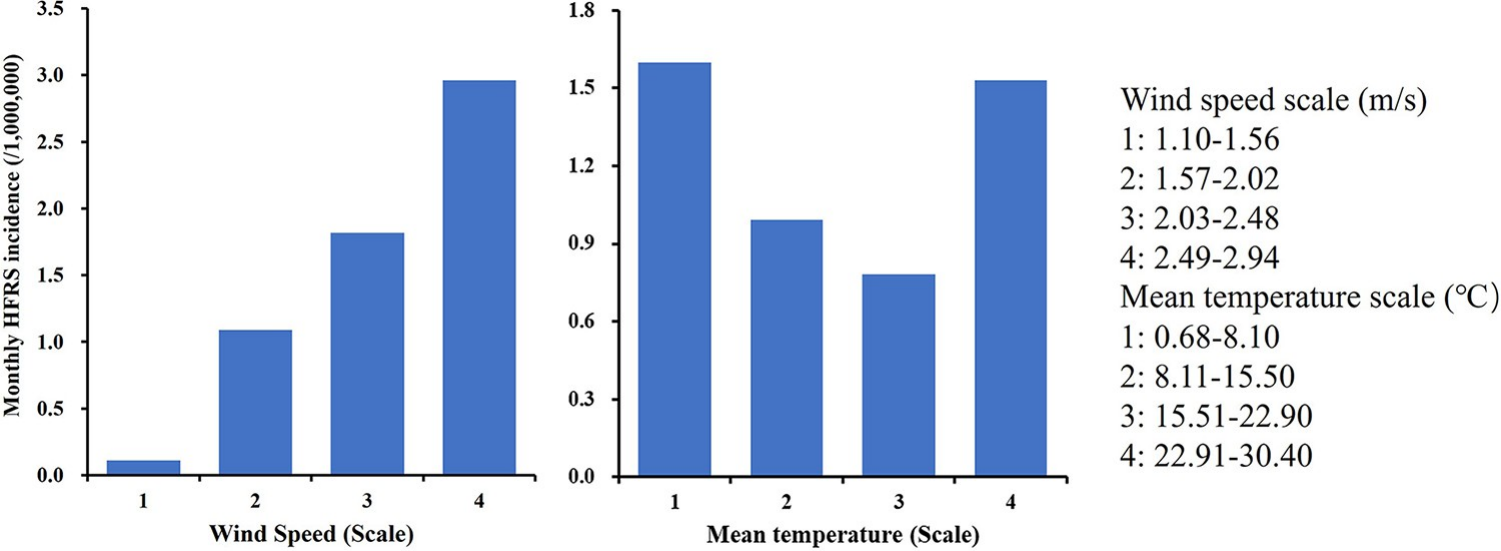
Effects of wind speed and mean temperature on the incidence of HFRS in Hubei province, 2016–2020.

The information of monthly meteorological data of 17 cities in Hubei province was available during 2016–2020, so we tested the effects of these factors on SSH of HFRS at the city level in 2016–2020. In the current study, mean temperature, wind speed and precipitation were categorized to 4, 4, 3 groups based on equal intervals respectively. Relative humidity was classified into 3 groups based on geometrical interval. The results of the Geodetector model showed that wind speed (*q* = 3.21%) and mean temperature (*q* = 1.03%) were the significant determinants of HFRS (both *P* <0.05), while the relations between precipitation (*q* = 0.47%, *P* = 0.11), relative humidity (*q* = 0.44%, *P* = 0.11) and monthly HFRS incidence were not significant. As shown in **Fig 5**, the incidence of HFRS increased with the wind speed, with the mean incidence every month at 0.1/1,000,000 and 3/1,000,000 in lowest and highest wind speed conditions, respectively. There was a “V”-shaped relationship between mean temperature and HFRS risk. The monthly HFRS risk was lowest at 0.8/1,000,000 when mean temperature fluctuated between 15.5–22.9°C, and the monthly incidence increased to 1.6/1,000,000 and 1.5/1,000,000 in the lowest and highest mean temperature conditions, respectively.

### 3.4. Time series trend of HFRS

By constructing an ARIMA model (0, 0, 2) (1, 1, 1), a general stable trend of incident HFRS cases was predicted in Hubei province in the next 3 years (S1A Fig). S1B Fig shows the plots of ACF and PACF. The ACF and PACF of residuals were near zero and within the confidence intervals, indicating the residuals were randomly distributed. Results of Ljung-Box Q test showed that the residual series were white noise process (*P* = 0.52). The accuracy and performance of the model were summarized by MAPE (MAPE = 37.02%), MAE (MAE = 8.13 monthly cases), RMSE (RMSE = 11.86) and R^2^ (R^2^ = 0.70). A total of 1,223 HFRS cases were projected in Hubei province during 2023–2025, with a range of 10 to 66 monthly cases. Comparison of monthly HFRS cases observed in 2023 with that fitted by ARIMA model were presented in S1A Fig. The observed values in 2023 were lower than the fitted values, mostly falling within the confidence intervals.

## 4. Discussion

Our investigation is the first comprehensive study to explore the long-term spatiotemporal dynamic distribution of HFRS during 2005–2022 and quantify the effects of meteorological factors on HFRS in Hubei province, providing vital evidence for a better understanding of the HFRS epidemic.

We investigated systematically the temporal trend of HFRS in Hubei province during 2005–2022. The incidence remained fluctuations at a low level during 2005–2015. Previous studies reported the incidence of HFRS had a clear decreasing trend since the outbreak in 1983 in Hubei province, due to the synthetic public health policies for prevention and control, like immunization program and rodent control [5]. The improvement of sanitation and public health also contributed to the downward trend. The incidence increased markedly between 2016 and 2018. It may correspond to the epidemic periodicity of infectious disease [19]. Besides, it may be related to the ecological agriculture model of integrated rice-fish farming in Hubei province, which provided enough food for rodent survival. Previous studies reported that the area of rice fishing co-culture developed rapidly after 2014 [20], and the rat density increased in 2015 [9]. Since the outbreak in 2018, emergent vaccination was administered, especially in high-risk areas and susceptible populations. The incidence declined significantly, reminding us the decisive role of vaccination in the prevention of possible epidemics and HFRS control.

High prevalence of HFRS was concentrated in the central areas of Hubei province. The main endemic areas in the period 2005–2015 were located along the Yangtze River valley and the Han River valley. The crowd and farmland gathered here, because of the low altitude, plentiful water systems, and plenty of annual rainfall, which provided ideal habitats for rodent populations. The epidemic areas transmitted to the southern areas, mainly in counties in Jingmen and Jingzhou cities in the latter periods, partly coincided with the areas of rice fishing co-culture. Jingzhou and Jingmen cities were the two major areas of rice fishing co-culture, accounting for more than 50% of the total area in Hubei province [20]. The transmission of production and lifestyle increased rodent populations and the chance of contact between humans and rats.

Meteorological factors were reported to closely affect the risk of HFRS, by influencing rodent population dynamics and the consequent human-rodent interaction [10,21]. In the current study, we found that wind speed and mean temperature were associated with the risk of HFRS in Hubei province, with an explanatory power of about 3.21% and 1.03%, respectively. Wind speed was analyzed as a factor in some studies and most showed positive association with HFRS [22,23], consistent with our results. Wind velocity could resuspend the virus particles, increase the survival time of the virus, and promote the transmission, leading to increased infection of HFRS [24]. In the current study, lowest risk of HFRS was identified when mean temperature ranged from 15.51 to 22.90°C, below which there was an inverse association between mean temperature and HFRS, yet with higher mean temperatures, there was a positive association. Results of the association between temperature and HFRS risk were inconsistent and hard to collate, including positive [25–27], negative [23,28] and non-linear relations [24,29,30]. A study conducted in Tai’an city, Shandong province reported a similar non-linear association [29]. Ambient temperature influences the behaviors of both mice and human, meanwhile, affects the infectivity of virus [31,32]. As temperatures drop, temperature variation between indoor and outdoor increases, with the result that rodents enter human life areas closely for food [30]. Besides, low temperatures may favor the survival of virus outside the hosts [32]. High temperatures favor the survival rate and density of rodents, growth of vegetation and crop, human outdoors activities, totally hence increasing risk of infection [33]. The relations between precipitation, relative humidity and HFRS were not significant in this study. Previous studies in regard to above associations were inconclusive. There were several reasons for the differentiated results, including regional characteristics, climate types, and statistic models. Climate factors affect the risk of HFRS in multiple ways, and the effects varied in different areas, so more studies are needed to explore the effects of climate factors on risk of HFRS in Hubei province. The current results alert us that surveillance of HFRS cases as well as paying close attention to climate factors are needed, so that comprehensive strategies could be taken in advance.

Our study provides valuable insights into the spatiotemporal pattern across a long-term period and influencing factors of HFRS in Hubei province. There also were some limitations. Firstly, we did not distinguish types of HFRS, like Hantaan virus or Seoul virus, which may introduce uncertainties in results. Secondly, we explored the effects of meteorological factors at the city level and the study design was an ecological study, which may have an ecological fallacy effect and then limit the capacity of causal inference. Thirdly, our study focused on the effect of limited climate factors, and has not yet to include other vital factors influencing HFRS, like environmental factors, socioeconomic factors, rodent density, vaccine injection, and so on, which may affect the influence of meteorological factors on the incidence of HFRS in Hubei province. Future studies are warranted to investigate the impacts of the elements. Finally, Generalization of the results is limited due to the factors mentioned above.

## 5. Conclusion

This is a comprehensive study that explores the spatiotemporal distribution of HFRS in a long-term view, and identifies the effect of meteorological factors in Hubei province. The HFRS incidence experienced a fluctuating at low level-increasing-decreasing trend in Hubei province during 2005–2022. The high-risk areas were concentrated in the center of Hubei. It is necessary to strengthen surveillance, rodent control and vaccination in these high-risk areas, to prevent the occurrence of outbreaks. In Hubei province we found that meteorological factors could significantly affect the distribution of HFRS, especially the wind speed and mean temperature. The information provides a basis for further study and the exploration of early warning systems.

## Supporting information

S1 Fig ARIMA (0, 0, 2) (1, 1, 1) model fitting and prediction results. A. The fitting and prediction values. B. The plots of ACF and PACF.
(TIF)

S1 Text Code of Geodetector model and ARIMA model. (DOCX)
