# Supplementary figures and images for: Spatiotemporal distribution and meteorological factors of hemorrhagic fever with renal syndrome in Hubei province
Source: PLoS Negl Trop Dis. 2024 Nov 4;18(11):e0012498. doi: 10.1371/journal.pntd.0012498 (PMC11563435; doi:10.1371/journal.pntd.0012498)

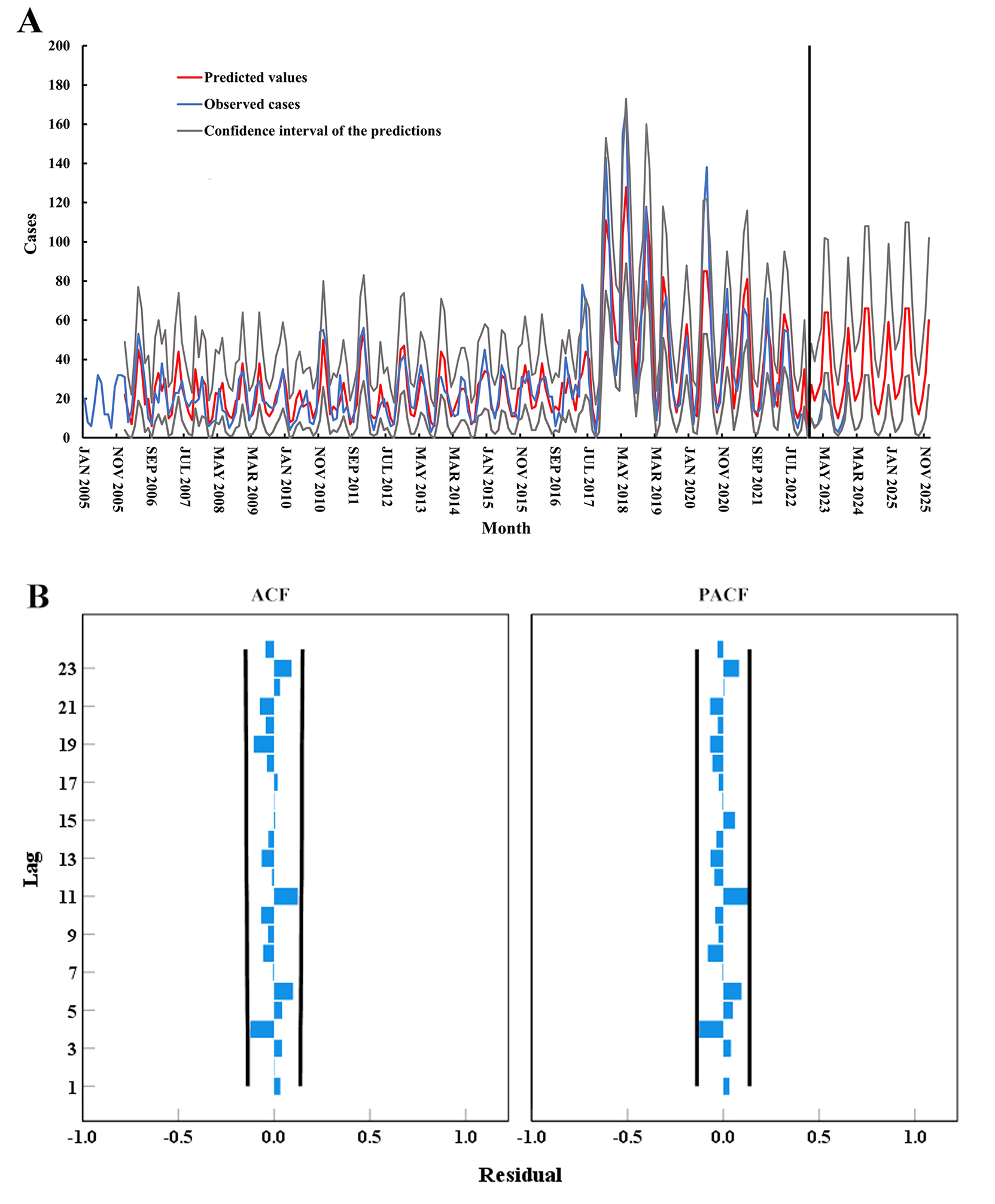

Supplement: S1 Fig — A. The fitting and prediction values. B. The plots of ACF and PACF. (TIF) [file pntd.0012498.s001.tif]
