# Supplementary material for: Spatiotemporal distribution and meteorological factors of hemorrhagic fever with renal syndrome in Hubei province
Source: PLoS Negl Trop Dis. 2024 Nov 4;18(11):e0012498. doi: 10.1371/journal.pntd.0012498 (PMC11563435; doi:10.1371/journal.pntd.0012498)
Supplement: S1 Text — (DOCX) [file pntd.0012498.s002.docx]

**The code of Geodetector model in R:**

install.packages("GD")

library("GD")

dataframe3 <- as.data.frame(rdata3)

discmethod <- c("equal","natural","quantile","geometric","sd")

discitv <- c(3:4)

datagdm3 <- gdm(incidence2018 ~ windspeed+rainfall+temperature+humidity,

continuous_variable = c("windspeed","rainfall"," temperature","humidity"),

data=dataframe3,discmethod = discmethod, discitv = discitv)

datagdm3

**The code of ARIMA model in SPSS:**

FILE='D:\data\ARIMA.sav'.

DATASET NAME dataset1 WINDOW=FRONT.

PREDICT THRU YEAR 2025 MONTH 12.

* TIMESERIESMODEL.

TSMODEL

/MODELSUMMARY PRINT=[MODELFIT]

/MODELSTATISTICS DISPLAY=YES MODELFIT=[ SRSQUARE RSQUARE RMSE MAPE MAE NORMBIC]

/MODELDETAILS PRINT=[ PARAMETERS RESIDACF RESIDPACF FORECASTS] PLOT=[ RESIDACF RESIDPACF]

/SERIESPLOT OBSERVED FORECAST FIT FORECASTCI

/OUTPUTFILTER DISPLAY=ALLMODELS

/SAVE PREDICTED(pre) LCL(LCL) UCL(UCL)

/AUXILIARY CILEVEL=95 MAXACFLAGS=24

/MISSING USERMISSING=EXCLUDE

/MODEL DEPENDENT=cases

PREFIX='MODEL'

/EXPERTMODELER TYPE=[ARIMA] TRYSEASONAL=YES

/AUTOOUTLIER DETECT=ON TYPE=[ ADDITIVE LEVELSHIFT].
